# Sarcoma risk and dioxin emissions from incinerators and industrial plants: a population-based case-control study (Italy)

**DOI:** 10.1186/1476-069X-6-19

**Published:** 2007-07-16

**Authors:** Paola Zambon, Paolo Ricci, Emanuela Bovo, Alessandro Casula, Massimo Gattolin, Anna Rita Fiore, Francesco Chiosi, Stefano Guzzinati

**Affiliations:** 1 Veneto Region, Assessorato alle Politiche Sanitarie, Istituto Oncologico Veneto, I.R.C.C.S. Veneto Tumour Registry, Via Gattamelata, 64, 35128 Padova, Italy; 2 Department of Oncological and Surgical Sciences, University of Padua, Via Gattamelata, 64, 35128 Padova, Italy; 3 Local Health Unit of Mantova, Unit of Epidemiology, Via Trento, 6, 46100 Mantova, Italy; 4 Province of Venice, Adviser, Department of Environment, Via Forte Marghera, 191- 30173 Mestre – Venice, Italy; 5 Province of Venice, Department of Environment, Via Forte Marghera, 191- 30173 Mestre, Venice, Italy

## Abstract

**Background:**

It is not clear whether environmental exposure to dioxin affects the general population. The aim of this research is to evaluate sarcoma risk in relation to the environmental pollution caused by dioxin emitted by waste incinerators and industrial sources of airborne dioxin. The study population lives in a part of the Province of Venice (Italy), where a population-based cancer registry (Veneto Tumour Registry – RTV) has been active since 1987.

**Methods:**

Two hundred and five cases of visceral and extravisceral sarcoma, confirmed by microscopic examination, diagnosed from 01.01.1990 to 31.12.1996, were extracted from the RTV database. Diagnoses were revised using the actual pathology reports and clinical records. For each sarcoma case, three controls of the same age and sex were randomly selected from the population files of the Local Health Units (LHUs). The residential history of each subject, whether case or control, was reconstructed, address by address, from 1960 to the date of diagnosis. All waste incinerators and industrial sources of airborne dioxin in the Province of Venice were taken into account, as was one very large municipal waste incinerator outside the area but close to its boundaries. The Industrial Source Complex Model in Long Term mode, version 3 (ISCLT3), was used to assess the level of atmospheric dispersion. A specific value for exposure was calculated for each point (geo-referenced address) and for each calendar year; the exposure value for each subject is expressed as the average of specific time-weighted values. The analysis takes into account 172 cases and 405 controls, aged more than 14 years.

**Results:**

The risk of developing a sarcoma is 3.3 times higher (95% Confidence Interval – 95% CI: 1.24 – 8.76) among subjects, both sexes, with the longest exposure period and the highest exposure level ; a significant excess of risk was also observed in women (Odds Ratio OR = 2.41, 95% CI: 1.04 – 5.59) and for cancers of the connective and other soft tissue (International Classification of Diseases, ninth Revision – ICD-IX 171), both sexes (OR = 3.27, 95% CI: 1.35 – 7.93).

**Conclusion:**

Our study supports the association between modelled dioxin exposure and sarcoma risk.

## Background

The emissions from incinerators and industrial plants contain various substances classed as certain or suspected carcinogens: metals, heavy metals, polyaromatic hydrocarbons (PAHs), polycyclic aromatics (PCA), dioxins (PCDDs and PCDFs).

Polychlorinated dibenzo-*p*-dioxins (PCDD) and polychlorinated dibenzofurans (PCDF), commonly known as dioxins, are pollutants that are mostly generated by human activity. The main sources are combustion, metal smelting, refining, and processing, and chemical manufacturing and processing.

The most toxic of these compounds, which persist in the environment and bioaccumulate, is 2,3,7,8 tetrachlorodibenzo-*p*-dioxin (TCDD).

The two main routes through which dioxins enter the food chain and human diet are the following: air-plants-animals and water-sediments-fish.

In 1997, the International Agency for Research on Cancer (IARC) classified TCDD as a Group I carcinogen on the basis of limited evidence in humans, sufficient evidence in animals and the consideration that the Ah receptor, through which dioxin acts, is present in both humans and animals [1]. The epidemiological evidence in humans was taken from 4 cohort studies carried out on subjects occupationally exposed to high levels of dioxin and from a study on the resident population of Seveso (Milan, Italy).

This evaluation was strongly criticised by Cole et al. [2] who, including in their analysis the data found in the literature after 1997, held that scientific evidence supported, if any, the hypothesis that TCDD was not a carcinogen. On the contrary, according to Steenland et al. [3], the new epidemiological and toxicological evidence reinforced the IARC's assessment and indicated that levels of exposure closer to those involving the general population can be carcinogenic.

The U.S. Environmental Protection Agency (EPA), which in 1985 had classified TCDD as a "probable human carcinogen" based on the data available at the time, concluded in the 2003 Reassessment [4] that TCDD was "best characterized as carcinogenic to humans".

In 2006, the National Research Council committee in charge of revising the *EPA draft Reassessment of the risks of dioxin and dioxin-like compounds*, unanimously concluded, based on the updating of carcinogen risk assessment guidelines, that TCDD should be classified "at least" as "likely to be carcinogenic to humans" [5].

Epidemiological studies indicate an increase in risk for tumours at all sites for high levels of exposure, while few and heterogeneous results are available concerning the effects in humans of long exposures at low levels, as well as at levels that the general population may be exposed to. In the Veneto region (North East Italy, 4,527,694 inhabitants at the 2001 census) a population tumour registry (Veneto Tumour Registry, RTV) has been active since 1987, covering the resident population in 15 of the 36 Local Health Units (LHUs) in which the region is divided. After the RTV reported an excessive risk of sarcoma in the period 1990–1996, the Regional Department for Prevention asked the RTV to carry out, in three LHUs within the Province of Venice, with the collaboration of the Municipality and the Province of Venice, an analytical study on dioxin exposure and sarcoma risk in this area.

## Methods

### Study area

**Figure 1:**
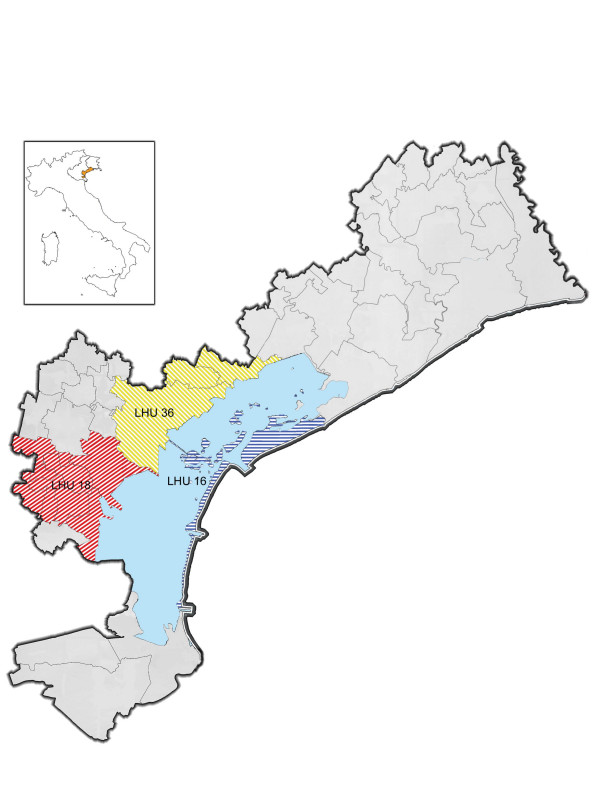
The coloured areas indicate the LHUs studied; (small box: Italy, Veneto Region, province of Venice).

The three LHUs involved in the study cover about one third of the Province of Venice, and the population of this area is about half of the provincial population (422,924 subjects, 2001 census), (Figure 1). The area includes the historical centre of Venice (LHU 16), the Venice mainland with the industrial area of Porto Marghera (LHU 36) and 9 Municipalities along the Brenta river (LHU 18).

Porto Marghera was the first Italian industrial area and its main plants are: a petrochemical industry (mainly chlorinated compounds), an oil refinery, industrial plants for the production and transformation of non-ferrous metals (aluminium, copper, and zinc), thermal power plants, waste incinerators. In the LHU of the Riviera del Brenta the main sectors are shoe manufacturing and steelworks, with limited agricultural production.

The rest of the provincial territory is not covered by the RTV.

### Selection of cases and controls

All histologically-confirmed cases of malignant sarcoma in all age groups and sites detected in the period between 1 January 1990 and 31 December 1996 were extracted from the RTV database, using the following International Classification of Diseases for Oncology, second edition (ICDO-II) morphology codes:

- M 880 – 892: not otherwise specified (NOS) sarcoma, fibrosarcoma, myxosarcoma, liposarcoma, myosarcoma;

- M 899: mixed mesenchymal sarcoma,

- M 904: synovial sarcoma

- M 912–913, M915 – 916: blood vessel sarcoma

- M 917: lymphatic vessel sarcoma

- M 954 – 957: nerve sheath sarcoma

- M 958: alveolar sarcoma

Mesotheliomas, Kaposi sarcomas, mixed forms and sarcomas with ICDO-II topography codes C 40–41 (bone) were excluded.

Diagnoses were revised by two RTV clinicians using the actual pathology reports and clinical records.

Two hundred and five cases met the criteria for inclusion and were resident at the time of diagnosis in one of the Municipalities of the 3 LHUs of the Province of Venice: 186 cases (90.7%) were confirmed by the revision. The diagnoses were classified according to the International Classification of Diseases, ninth Revision (ICD-IX). We used controls drawn from the general population files of the 3 LHUs studied, taking into account the life status and residence on 01.01.1990.

For each case, three individual matched controls were chosen at random among those of the same sex and age at the time of diagnosis. However, we verified that controls were not already in the RTV database with a diagnosis of sarcoma

### Reconstruction of residential history

The residential history of each subject (186 cases and 558 controls) was reconstructed address by address for the period from 1960 to the date of diagnosis. Information was gathered from the Population Registries of each of the Municipalities where the subject had lived during the period studied. Each address, (total 1,823) in the Province was then geo-referenced, using the cartographic reference system for Italy (Gauss Boaga Projection) [6]. Only five addresses turned up to be non-existent as they are due to identity protection measures.

### Exposure data

A survey of the incinerators and industrial sources of airborne dioxin in the Province of Venice was carried out. A very large municipal waste incinerator in the neighbouring Province of Padua was also considered, because of its being very close to the boundary of the area under study.

Thirty-three plants were taken into consideration: 4 Industrial Waste Incinerators (IWI), 10 Municipal Solid Waste Incinerators (MSWI), 12 Medical Waste Incinerators (MWI), 3 thermal power plants, 1 oil refinery and 3 industrial plants for the production of primary aluminium.

Emission levels were calculated through a historical reconstruction of the technology used by each plant and the quantity and quality of the waste/refuse treated.

The analysis started off by using a series of indicators, taken from different sources, to define the point of emission peculiarity (high mass flow rate emissions and others) and emission factors in reference to the type of process and pollution reduction technologies applied.

**E**_**i **_= **A **× **EF**_**i**_

with:

▪ E_i _= mass flow of i – pollutant emitted (kg/year);

▪ A = activity key parameter, e.g.: raw material consumption, fuel used, final product obtained (ton/year);

▪ EF_i _= Emission factor for PCDD/PCDFs (kg_pollutant_/ton_fuel._).

The historical rating of dioxins released over the past 40 years required both a bibliographical and historical study of process analysis to collect data on raw material consumption, start up, changes, improvements, and closing down of plants, as well as pollution reduction systems.

This kind of analysis provided awareness on process layout and changes spanning the last 40 years and also led to an inquiry into the characteristics of pollutant treatment systems.

For industrial sources of PCDD/PCDF emissions, location and operational data needed to calculate emissions were taken from the administrative documentation deposited in the offices of the Province of Venice, the agency in charge of control in this sector, and from the proceedings of an important lawsuit concerning the pollution caused by the petrochemical plant of Porto Marghera (Venice) [7].

With regards to solid urban waste, data were taken from the administrative documentation of the Province of Venice and also from technical surveys carried out at plant sites. The documentation was integrated with interviews and questionnaires to engineers of the plant managing companies. With regards to hospital incinerators, data were taken from the administrative documentation of the Veneto Region, which carried out a survey of these incinerators in 1984 in order to plan their gradual shutdown.

It was thus possible to identify the period when each plant was active and the type of incinerator with acceptable accuracy. The quantity of waste disposed was calculated on the basis of parameters provided in the documents of the Veneto Region: number of beds; their level of occupation, amount of waste per patient per day, divided into assimilable or non assimilable to urban waste.

The Industrial Source Complex Model in Long Term mode, version 3 (ISCLT3) model, developed by the US EPA, was used to assess the level of atmospheric dispersion of the polluting substances; the model takes into consideration wind speed and direction and the degree of atmospheric stability which causes fog to form [8].

A specific value for exposure was calculated for each point (geo-referenced address); the value for each address in a given year is the sum of the values calculated for the plants that were active during that year and were located within a 50 kilometre radius. The exposure value for each subject is the average of the values of the single addresses, weighted by time, i.e., by the number of days the subject lived at that specific address. This exposure value was expressed in WHO TEQ (PCDD/PCDFs), using 1998 Toxic Equivalency Factors (TEF) values [9].

**Figure 2:**
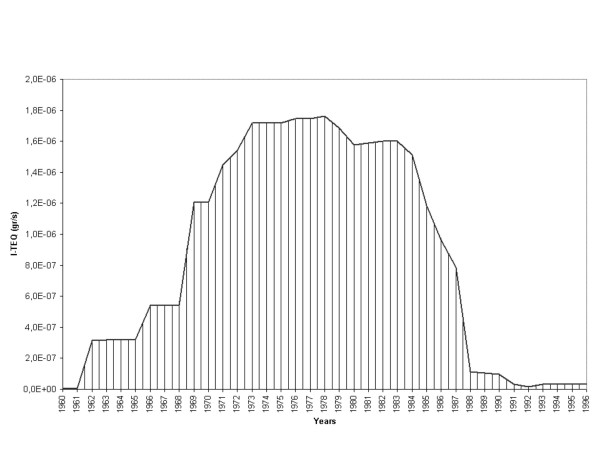
Trend by year of the emission levels of the incinerators and industrial plants (I-TEQ gr/s).

Figure 2 shows a graph of emission levels over time; the value of each year was obtained by adding the emissions of all the plants active that year. The peak was reached in the period 1972 – 1986, after which emissions returned to their former level.

### Population on analysis

Seventeen controls out of 558 were lost because the Municipal Population Registers showed that by 1990 they were either no longer resident or had died.

The following subjects were also excluded from the analysis:

- 3 childhood cases, born after 1986 when the exposure studied had ceased and their 9 matched controls;

- 2 cases, correlated with recognised risk factors: one radiation-induced case and one associated with von Recklinghausen's disease [10] and their 6 matched controls;

- 9 cases of subjects who did not continuously reside in the Province or moved there after 1969; since it was not possible to assign a level of exposure to addresses in other provinces, in our study we only considered subjects who were already living in the area under study in 1969 or resided there from birth. We believe subjects could not be exposed before 1969 even if they resided elsewhere because Porto Marghera (Venice) was the first industrial area created in Italy after the Second World War and Veneto was also the place were the first incinerators for urban and hospital waste were built; we also excluded their 27 matched controls;

Moreover, among controls we also excluded the following:

- 59 controls with malignant tumours, all sites, registered in the RTV database, because dioxins are considered to be carcinogens for all types of cancer: only cases of non melanoma skin cancer were accepted;

- 35 controls who were not continuously resident in the Province or had moved there after 1969. Some controls were excluded for both reasons; in this case only the first reason given in the list above counted. Therefore people without malignant tumours, residing in the Province since 1960 (beginning of the exposure), or having moved there before 1970 (since exposure is unlikely in other Provinces before 1970), or born in the Province before 1986 (end of the exposure period) were included as controls. The population of the study was made up of 172 cases (92.5%) and 405 controls (72.6%).

We also performed a sensitivity analysis only on subjects that had permanently resided in the area since 1960 or resident from birth (168 cases and 384 controls).

### Statistical analysis

Analyses were carried out on the total study population, divided into three classes of average exposure and two classes of length of exposure, as well as by sex and the following ICD-IX codes: ICD-IX 171 (malignant neoplasm of connective and other soft tissue), ICD-IX 158 (malignant neoplasm of retroperitoneum and peritoneum), ICD-IX 173 (malignant neoplasm of skin other than melanoma of skin, Kaposi's sarcoma and skin of genital organs) and all other codes (visceral sites).

We fitted a quadratic logistic regression spline model to estimate the Odds Ratio curve as a function of the level of exposure used as continuous variable; the values of the points at which the curve slope changed were rounded and taken as cut-off points (4 and 6 fg/m^3^) (11,12). For the exposure length classes, we considered the approximate median value. Sarcoma risks by sex and by ICD-IX code were only analysed for level of exposure because the time-period variable was not significant. We used conditional logistic regression to calculate Odds Ratio (OR) values and 95% Confidence Interval, and the Wald chi-square test for trend, using the SAS Software [13,14].

In addition, to examine the spatial distribution of subjects, we also performed a cluster analysis with a Bernoulli model, applying spatial scan statistic with 999 Monte Carlo replications and 50% of the total study population as maximum cluster size [SatScan Software,15]. In this analysis, for every subject, whether case or control, we only considered the geographical location of the address where he/she was resident in the peak period for emissions (1972–1986). When a subject had lived at more than one address in that period, then the address at which he or she had lived for the longest period was taken into account (prevalent address). Length of period of residency was greater than 10 years out of 15 for 86% of subjects and greater than 13 out of 15 years for 67%.

## Results

The first step in our analysis was to calculate the risk among the population that had permanently resided in the area since 1960 or, for younger subjects, was resident from birth: this population included 168 cases and 384 controls with a median exposure value of 4.22 fgr/m^3 ^and a median length of 32.84 years. Analysis by quartiles of the population, with cases and controls taken together, revealed an OR value of 1.86, with 95% CI: 1.11 – 3.13 in the most exposed quartile. This OR value is almost the same as that related to the most exposed quartile calculated on a population of 172 cases and 405 controls, which includes the 25 subjects (4 cases and 21 controls) not resident at 01.01.1960 but who moved to the Province of Venice before 01.01.1970: OR = 1.91, 95% CI: 1.14 – 3.19.

Further analysis was then carried out on this population of 172 cases and 405 controls. In this population, the median exposure value for cases and controls taken together was 4.25 femtogramms/m^3 ^and the median value of length of exposure was 32.74 years.

Table 1 shows the distribution of cases and controls in relation to the three levels of average exposure and two classes of length of exposure, with the corresponding OR values and Confidence Interval (95% CI). Risk increases in relation to both the duration and the extent of exposure and is statistically significant in the class with longest period and highest level of exposure (OR = 3.30, 95% CI: 1.24 – 8.76).

**Table 1:** ORs and 95%CI of sarcoma by length and levels of exposure (both sexes, all sites)

	**Average exposure (fgr/m^3^)**		
	
**Length of exposure (years)**	< 4 Cases/Controls OR (95% CI)	4–6 Cases/Controls OR (95% CI)	≥ 6 Cases/Controls OR (95% CI)
< 32	10/41 1*	41/100 **1.67** (0.76 – 3.68)	14/26 **2.57** (0.95 – 6.92)
≥ 32	45/120 **1.61** (0.71 – 3.63)	42/92 **1.91** (0.84 – 4.34)	20/26 **3.30** (1.24 – 8.76)
Total	55/161 1*	83/192 **1.27** (0.84 – 1.91)	34/52 **2.08** (1.19 – 3.64)

* Reference category

In both sexes, risk increases in relation to the level of exposure but reach statistical significance only for women, with an OR of 2.41 among the most exposed (95% CI: 1.04 – 5.59) and a significant test for trend (p < 0.04) (Table 2).

**Table 2:** ORs and 95%CI of sarcoma (all sites) by sex and levels of exposure

	**Average exposure (fgr/m^3^)**			
		
**Sex**	**<4** Cases/Controls OR (95% CI)	**4–6** Cases/Controls OR (95% CI)	**≥ 6** Cases/Controls OR (95% CI)	**χ_1_^2 ^for trend**
**Males** (Ca/Co: 87/197)	31/83 1*	39/88 **1.1** (0.63 – 1.96)	17/26 **1.86** (0.87 – 3.95)	2.05 (p = 0.1517)
**Females** (Ca/Co: 85/208)	24/78 1*	44/104 **1.47** (0.82 – 2.66)	17/26 **2.41** (1.04 – 5.59)	4.30 (p = 0.0382)

* Reference category

Table 3 shows analysis for ICD-IX code for both sexes. In the most exposed cases, with ICD-IX 171 code (connective and other soft tissue) there is a significant risk excess (OR = 3.27, 95% CI: 1.35 – 7.93); risk increases for visceral sites, as well (OR = 2.45, 95% CI: 0.96 – 6.28), while there is no evidence of risk for sarcomas in peritoneal/retroperitoneal (ICD-IX 158) and skin sites (ICD-IX 173).

**Table 3:** ORs and 95%CI of sarcoma by ICD-IX code^1 ^and levels of exposure, both sexes

	**Average exposure (fgr/m^3^)**			
		
**ICD-IX code**	**<4** Cases/Controls OR (95% CI)	**4–6** Cases/Controls OR (95% CI)	**≥ 6** Cases/Controls OR (95% CI)	**χ_1_^2 ^for trend**
**ICD-IX 171** (Cases/Controls: 81/190)	25/80 1*	39/93 **1.35** (0.73 – 2.48)	17/17 **3.27** (1.35 – 7.93)	5.89 (p = 0.0152)
**ICD-IX 173** (Cases/Controls: 17/43)	5/12 1*	10/20 **0,04** (0.31 – 4.71)	2/11 **0.34** (0.03 – 3.43)	0.51 (p = 0.4758)
**ICD-IX 158** (Cases/Controls: 21/49)	6/14 1*	12/27 **1.06** (0.33 – 3.43)	3/8 **0.8** (0.14 – 4.45)	0.03 (p = 0.8625)
**Visceral sites** (Cases/Controls: 53/123)	19/55 1*	22/52 **1.24** (0.60 – 2.55)	12/16 **2.45** (0.96 – 6.28)	3.02 (p = 0.0823)

* Reference category

^1^ICD-IX 171: malignant neoplasm of connective and other soft tissue;

ICD-IX 173: malignant neoplasm of skin (other than melanoma of skin, Kaposi's sarcoma and skin of genital organs);

ICD-IX 158: malignant neoplasm of retroperitoneum and peritoneum.

The spatial scan statistic identified the most likely cluster for the Bernoulli model in four neighbouring Municipalities, all covered by LHU 18 (Riviera del Brenta). The spatial cluster consisted of 19 cases whereas 8.35 were expected; the Observed/Expected ratio is 2.28, statistically significant (p = 0.048).

## Discussion

The results of our research clearly show a significant increase in the risk of sarcoma, correlated both with the level and the length of environmental modelled exposure to dioxin-like substances. The risk excess is also evident in females, and, for both sexes taken together, for cancers of the connective and other soft tissue (ICD-IX 171).

A few observations should be made here regarding methods.

We believe that cases have been correctly and completely identified, as they are incident cases taken from a permanent tumour registry (RTV) that has been active for many years and whose data have been published since 1987 in the "Cancer Incidence in Five Continents" publications of the International Agency for Research on Cancer, Lyon [16,17].

By consulting the actual pathology reports and clinical records gathered by the RTV we were able to identify mixed forms (carcinosarcomas), sarcomas with "bone" topography, "possible" diagnoses, and cases associated with other known risk factors.

We used the LHU population files in order to obtain our control population. We found a few errors regarding municipality of residence and vital status in some of the records for the period. However, reconstruction of each subject's residential history on the basis of local municipal archives allowed us to retrospectively eliminate those subjects who were not resident in the areas studied on 01.01.1990, or who had died before then. About 10% of the controls were excluded from the study because they were affected by malignant cancer (all sites) registered in the RTV database; 6% of the controls were excluded on grounds of residency, a number comparable to the 4.8% of the cases excluded for the same reason. The final ratio of cases to controls was 2:3 for men and 2:4 for women.

The most complex methodological question concerns how dioxin exposure was calculated.

The ISCLT3 dispersion model requires the following meteorological variables: wind direction, speed and frequency; classes of atmospheric stability and vertical remixing. The only source that could provide this information for the period under study were the weather records from the Venice airport. They were used to calculate the emissions over the entire area and this could mean that estimates for the more distant plants are less accurate. However, we feel that this method does offer a fairly good representation of exposure. Cluster analysis identifies the highest risk area as 4 adjoining municipalities on the Brenta Riviera, and we note that this is also the area with the addresses that have the highest exposure levels, which is consistent with the prevalent wind direction.

Furthermore, the excess risk given by cluster analysis is close to the value of the Standard Incidence Ratio (SIR) calculated by the RTV for incident cases of sarcoma with ICD-IX 171 code in the period 1990 – 1996 in the Riviera del Brenta LHU. The SIR value was 1.82 (95% CI: 1.13 – 2.69) for males and 2.28 (95% CI:1.44 – 3.30) for females [18].

In 2003, a comparison was made between the modelled and monitored concentrations of three polluting substances (SO2, PTS, Nox); the agreement between the two different values for SO2 was more than satisfactory while for PTS and Nox which, unlike SO2, are not mainly of industrial origin, there was a larger difference [19]. However, because there are no measurements of dioxin levels available for the period studied, we cannot check our estimates against historical samplings. We evaluated other hypotheses of risk factors as alternatives or concurrent to the environmental pollution considered here: factors such as eating habits and occupational exposure.

The three LHUs cover a relatively small population (423,000 residents) and there is no reason to suppose that the eating habits of the cases are very much different from those of the controls, or that those of the people living on the Riviera del Brenta (inland) should be so very different from those of the Venetian lagoon dwellers. Furthermore, a recent study carried out by the Veneto region to monitor the level of dioxins and PCBs in foodstuffs (fish, meat, eggs, milk) suggested that the highest levels of these substances are found in shellfish, which are probably eaten more often in the lagoon area [20]. However, we have no specific information on consumption of local animal and plant products that could have been more highly contaminated by PCDD/PCDFs emitted by the incinerators and industrial plants.

We have no information about social status and only partial knowledge regarding occupation, nevertheless we consider it unlikely for occupational exposure to have had much influence, since to our knowledge, there were no industries in the area at the time where there would have been risk of exposure to dioxins. As regards cases between 35 – 69 years, the names of the firms and the industrial sectors that employed the private sector workers still active in 1974 are recorded in the electronic database of the Italian National Institute for Social Security (INPS) [21]. Only 35 subjects were found in this list, none of whom would appear to have worked in areas of production where there was a risk of exposure related to sarcoma. Subjects over 70, thus born prior to 1926, had plausibly retired from work by 1976, since the retiring age at the time was 50. If the source of exposure had been occupational, the latency time would have been extremely long, making this hypothesis unlikely.

Instead, the time elapsed between the exposure under study (1972 – 1986) and sarcoma diagnosis is compatible with the latency time of the carcinogenic effects of dioxin both in older and younger subjects. The study population had been widely exposed: 40% had lived at an address that was less than two kilometres and 88% within 5 kilometres from an incinerator or industrial plant.

Lastly, the significant excess of risk observed in women was unlikely to be due to occupational exposure, being instead primarily attributable to environmental exposure, given that women were less mobile in the past and would have rarely been subject to risk of occupational exposure to the pollutants studied.

A recent review of the literature on epidemiological studies concerning the effect on health of exposure to emissions from waste incinerators showed that a significant association between exposure and cancer was made in two thirds of the studies published by 2003 [22]; the strongest evidence of an association is in lung cancers, cancer of the larynx and non-Hodgkin lymphoma.

Exposure to dioxin has been associated with an increased risk of sarcoma [23-25] but the results of the studies are not yet conclusive. A recent study [26], carried out in Finland, examined 110 cases of soft tissue sarcoma and 227 hospital controls; exposure to dioxin was measured using the concentrations found in sub-cutaneous fat samples and risk did not increase with exposure; rather, the lowest level showed the highest risk in all types of analysis.

So far, very few studies have analysed the relationship between risk of sarcoma and emissions from incinerators. In 2000, Viel et al. [27] identified a cluster of sarcomas and non-Hodgkin lymphomas in a population living near a municipal waste incinerator, with high levels of emissions, at Besançon, France. Later studies have confirmed the result for non-Hodgkin lymphomas [28], but not for sarcomas [29]. Excess risk for non-Hodgkin lymphomas was only present in the area with the highest estimated concentrations of dioxin.

Comba et al. [30] reported a significant increase in the risk of sarcoma associated with living within a two kilometre radius of an incinerator burning industrial waste. The cluster is remarkable for the net prevalence of women among the cases: given that these women would not have been exposed to risk for occupational reasons, there can be no other explanation apart from the proximity of their home to the incinerator and excess risk does not extend beyond the 2 kilometre radius.

In order to define the level of exposure, the two studies carried out in France on clusters of non-Hodgkin lymphomas and sarcomas used a Gaussian type dispersion model which highlighted wind direction when identifying areas with different levels of pollution and gave a geographical representation of pollution. The same authors also conducted a further study in order to validate their method, using soil samples. On flat terrain they discovered a significant association between their estimated dioxin concentrations and the log-transformed measured dioxin soil concentrations, while in the more topographically complex areas their model tended to overestimate concentrations [31].

We used an analogous dispersion model and from the topographical point of view the entire Province is completely flat, so our estimates probably do provide a good estimate of dioxin exposure.

## Conclusion

Epidemiological evidence of the carcinogenic effect of dioxins is essentially supported by studies carried out on populations exposed to high levels of dioxins due to occupation or accident.

We believe that the results of our study support the association between modelled dioxin exposure and sarcomas in a general population exposed for a prolonged period of time to what are, in all likelihood, much lower concentrations.

## Competing interests

The author(s) declare that they have no competing interests.

## Authors' contributions

PZ is the principal investigator of this study. As such she participated in the design, planning, data analysis and writing of the present paper.

PR contributed equally to this work.

EB created the SAS archive of the residential history of the subjects and carried out the geo-referencing of addresses.

AC, MG and FC gathered analytical information on the incinerators and estimated the values of exposure.

ARF contributed to the revision of the diagnoses.

SG performed the statistical analysis.

All authors read and approved the final manuscript.
